# Brief Report: Central Nervous System Metastases After Chemoradiation Followed by Durvalumab for Unresectable Locally Advanced Nonsmall Cell Lung Cancer

**DOI:** 10.1016/j.cllc.2025.07.002

**Published:** 2025-07-05

**Authors:** John Sharp, Philip Young, Songzhu Zhao, Lai Wei, Sandip H. Patel, Mingjia Li, Jeremy Brownstein, Karl Haglund, Joshua Palmer, Raju Raval, Sasha Beyer, Logan Roof, Peter Shields, Kai He, Jacob Kaufman, Regan M. Memmott, Asrar Alahmadi, David P Carbone, Gregory A Otterson, Carolyn J Presley, Ryan D. Gentzler, Dwight H. Owen

**Affiliations:** a Division of Medical Oncology, The Ohio State University, Arthur G. James Cancer Hospital and Richard J. Solove Research Institute, Columbus, OH; b Division of Hematology and Oncology, Kaiser Permanente Tacoma Medical Center, Tacoma, WA; c Division of Hematology/Oncology, Adena Health System, Adena Cancer Center, Chillicothe, OH; d Division of Radiation Oncology, The Ohio State University, Arthur G. James Cancer Hospital and Richard J. Solove Research Institute, Columbus, OH; e Division of Hematology and Oncology, Department of Medicine, University of Virginia Cancer Center, Charlottesville, VA

## Introduction

Lung cancer is the most common cancer and cause of cancer death worldwide.^1^ At diagnosis, 20%−35% of patients with non-small cell lung cancer (NSCLC) have locally advanced (LA) disease.^2^ Patients with LA-NSCLC who are not surgical candidates are generally offered chemoradiation (chemoRT) followed by durvalumab per the PACIFIC trial (if *EGFR* and *ALK* negative). PACIFIC demonstrated an overall survival (OS) benefit,^3,4^ though with an 18-month progression-free survival of 44.2%, many patients are not cured.

Among these patients, a devastating pattern of recurrence is central nervous system (CNS) metastases. In patients with NSCLC with CNS metastases, median OS is 3.7–6.0 months.^5,6^ Long-term follow up of PACIFIC demonstrated CNS metastases in 6.5% of patients receiving durvalumab.^4^ Real-world rates may be higher.^7^ Improvements in management strategies^8–11^ have led to better outcomes for patients who develop CNS metastases, though no consensus on CNS surveillance imaging exists. Post-chemoRT CNS imaging is not routinely performed, though it could represent an opportunity to adjust treatment paradigms and intervene earlier if CNS metastases are found. Identification of those at highest risk for CNS metastases is needed to inform clinicians and patients, and to guide future clinical trial enrollment. Hence, we sought to describe incidence of, risk factors for, and survival associations with CNS metastases in patients with LA-NSCLC undergoing chemoRT followed by durvalumab.

## Materials and Methods

We conducted a retrospective study of consecutive patients with unresectable LA-NSCLC treated with chemoRT followed by durvalumab at 2 academic institutions between January 2018 and July 2022. This study was approved by the institutional review board of both centers.

### Patient Selection

Patients were ≥18 years, had histologically confirmed unresectable LA-NSCLC, no evidence of CNS metastases at baseline, and received concurrent chemoRT without progression followed by durvalumab. Patients with locally recurrent disease were included. Patients initially treated for oligometastatic disease were excluded. All patients completed staging with PET scan and brain MRI prior to chemoRT to exclude metastatic disease as standard of care. Following chemoRT, repeat CT chest was obtained to rule out progression and pneumonitis. Repeat CNS imaging was obtained in cases of symptoms suggestive of CNS metastases as there is no formal CNS monitoring recommended in National Comprehensive Cancer Network (NCCN) guidelines; scheduled CNS monitoring was also not performed in the PACIFIC protocol^3^ or similar PACIFIC-based real-world studies.^12,13^

### Data Collection

Baseline clinical characteristics were recorded. Staging brain MRI and subsequent imaging were reviewed to determine incidence of CNS metastases and systemic recurrence. Dates of CNS metastases and death were collected, as applicable.

### Outcomes

Primary outcome was factors associated with development of CNS metastases. Secondary outcomes included incidence of CNS metastases, time to development of CNS metastases (from initiation of durvalumab, censoring at death or last follow-up), and OS (from initiation of durvalumab, censoring those alive at last follow-up).

### Statistical Considerations

Baseline characteristics were summarized using means (standard deviations), medians (interquartile ranges), or frequencies (proportions), as appropriate. Cox proportional hazards models were used to identify potential risk factors for CNS metastases. Statistically significant (*P* < .1) and clinically relevant (age) variables in univariate analysis (UVA) were included in the multivariable analysis (MVA) with adjusted hazard ratios and 95% confidence intervals (CI) reported. As global stage is assessed at diagnosis, we explored the association between stage and risk of CNS metastases only in the subgroup of patients with *de novo* (not locally recurrent) LA-NSCLC. Kaplan-Meier method was used to estimate survival functions with log-rank test for inter-group comparisons. Analyses were conducted using SAS 9.4.

## Results

### Baseline Characteristics

In total, 193 patients were included with median follow-up of 25 months (IQR: 12–37). Median age was 64 years (range: 37–86), 85 patients (44.0%) were female, 96 (49.7%) had squamous histology, 155 (80.3%) had *de novo* unresectable LA-NSCLC while 38 (19.7%) had locally recurrent disease. Of patients with *de novo* disease, 73 (47.1%) had stage IIIA, 57 (36.8%) stage IIIB, 16 (10.3%) stage IIIC, and 9 (5.8%) had incomplete staging data. Genomic profiling was performed in 147 (76.6%) and identified mutations in *KRAS* in 29 (15.0%)*, TP53* in 57 (29.5%), and *STK11* in 8 (4.1%). Notably, 1 (0.5%) patient had *EGFR* exon 20 indel, S768_D770dup and none had *ALK* rearrangements. ChemoRT regimens were carboplatin-based in 171 (88.6%) with median number of weekly cycles of 6 (IQR: 5, 7), and cisplatin-based in 22 (11.4%), with median number of cycles of 2 (IQR: 1, 2). Most common radiation dosages were 60 Gy in 123 (63.7%), 66 Gy in 46 (23.8%) and other in 24 (12.4%). Baseline characteristics are summarized in supplemental Table 1.

### Risk Factors for CNS Metastases

Overall, 32 (16.6%) patients developed CNS metastases, 20 (62.5%) with isolated CNS metastases and 12 (37.5%) with synchronous systemic and CNS metastases. Median time to CNS metastases was 6.4 months (range: 1.4, 49.6). Overall, 78.1% (25/32) of CNS metastases occurred within 1 year of starting durvalumab.

**Figure 1:**
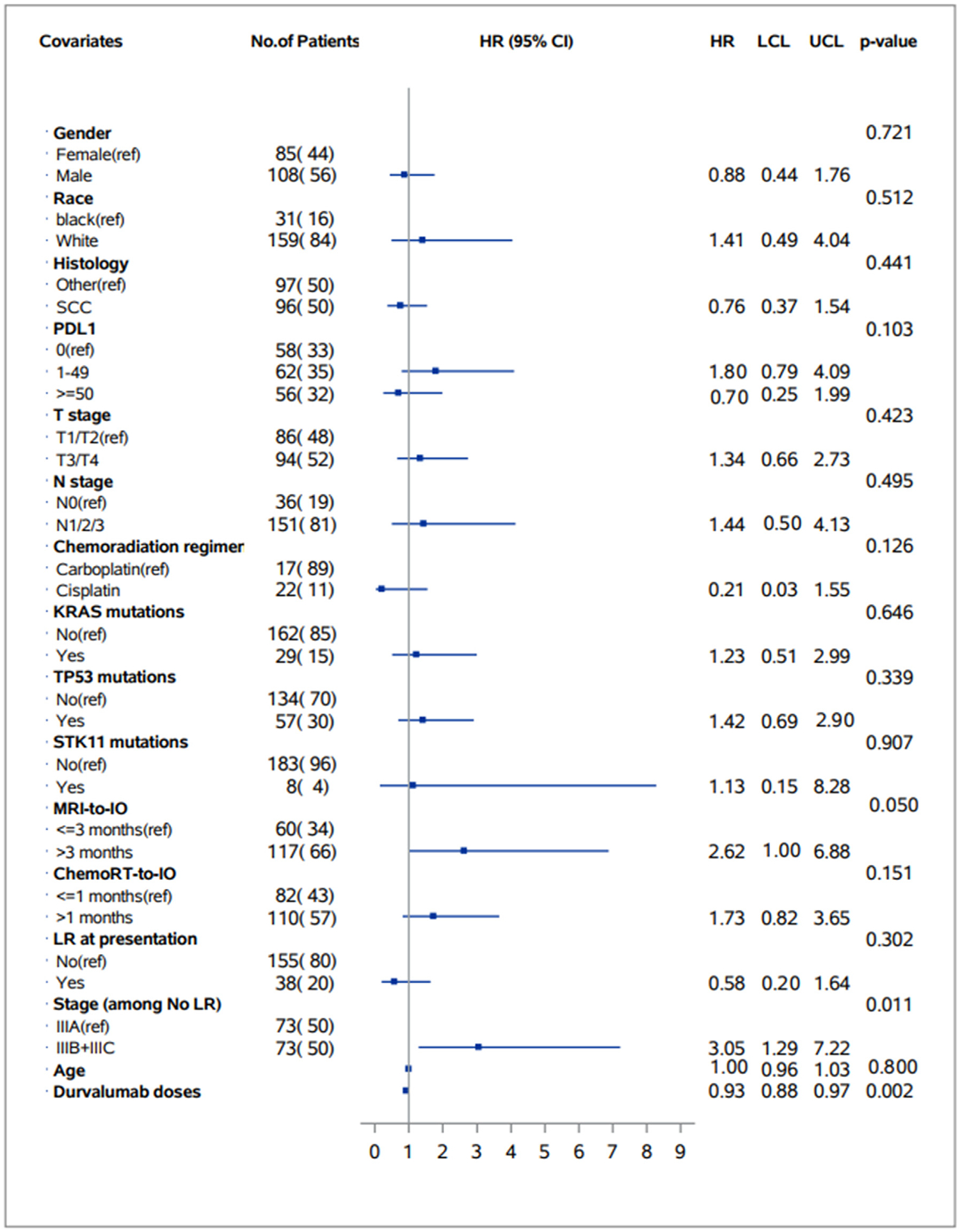
Forest plot of baseline clinical variables and association with risk of development of central nervous system metastases on univariable analysis. Abbreviations: chemoRT = chemoradiation; HR = hazard ratio; IO = immunotherapy; LCL = lower 95% confidence interval limit; LR = locally recurrent; MRI = magnetic resonance imaging; PDL1 = programmed cell death ligand 1; SCC = squamous cell carcinoma; UCL = upper 95% confidence interval limit.

Among patients with *de novo* LA-NSCLC, more advanced stage (IIIB/C vs. IIIA) was associated with increased risk of CNS metastases (HR 3.05 [95% CI: 1.29, 7.22] *P* = .01), otherwise no patient or disease-related variables associated with risk of CNS metastases (Figure 1). Other factors associated with CNS metastases included time from baseline brain MRI to initiation of durvalumab (MRI to IO interval) and number of durvalumab doses. Based on these findings, for the overall cohort (*n* = 193), we included age, MRI to IO interval, and number of durvalumab doses in the MVA. This revealed that MRI to IO interval >3 months was associated with a higher risk of developing CNS metastases (HR 2.66 [95% CI: 1,01, 7.02], *P* = .048). Furthermore, each additional dose of durvalumab was associated with a 7% decrease in risk of CNS metastases (HR 0.93 [95% CI: 0.89, 0.97], *P* = .003) (Table 1).

For the subgroup with *de novo* LA-NSCLC (*n* = 155), we included stage, MRI to IO interval, and number of durvalumab doses in the MVA. Again, longer MRI to IO interval and fewer durvalumab doses were associated with risk of CNS metastases. Additionally, increased overall stage (IIIB/C vs. IIIA) was associated with increased risk of CNS metastases (HR 3.85 [95% CI: 1.44, 10.35], *P* = .007) (Table 1).

### Survival Outcomes

**Figure 2:**
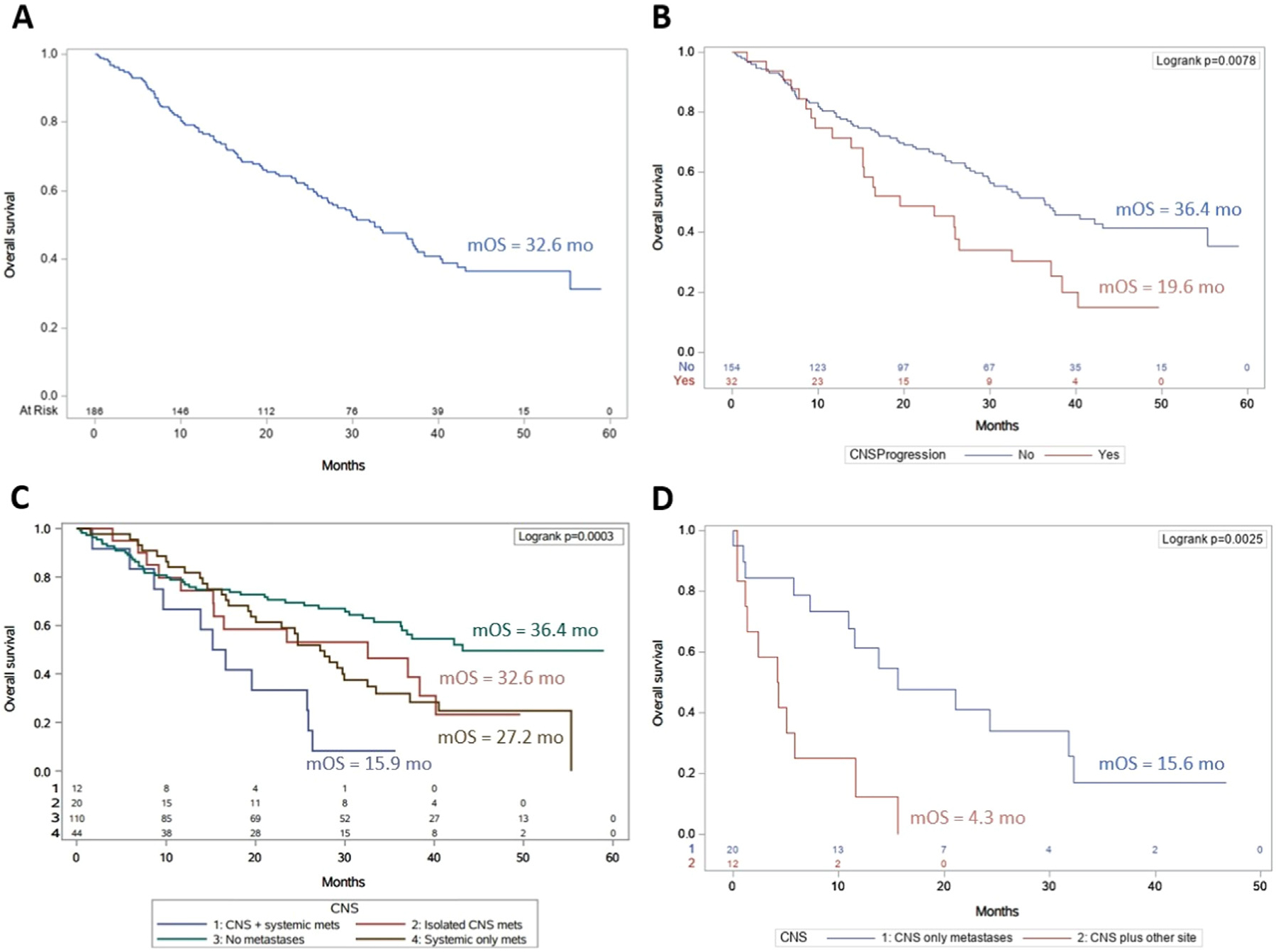
Overall survival (OS) curves for (A) all patients from start of durvalumab; (B) patients who developed central nervous system (CNS) metastases compared to those who did not; (C) patients with isolated CNS metastases vs. isolated systemic metastases vs. synchronous CNS and systemic metastases vs. no CNS metastases; and (D) isolated CNS metastases vs. synchronous CNS and systemic metastases. Abbreviations: CNS = central nervous system; *m* = median; mo = months; OS = overall survival.

Median OS was 32.6 months (95% CI: 27.1, 37.5) (Figure 2A). Patients who developed CNS metastases had shorter OS (median 19.6 months [95% CI: 13.9, 32.6] vs. 36.4 months [95% CI: 29.7, 55.3], *P* = .008, Figure 2B). Patients with synchronous systemic and CNS relapse had shorter OS compared to patients with isolated CNS relapse and those with isolated systemic relapse (median 15.9 months [95% CI: 5.9, 25.9] vs. 32.6 months [95% CI: 11.7, 40.2] vs. 27.2 months [95% CI: 19.2, 32.5], respectively, *P* < .001, Figurex 2C). Median OS from onset of CNS metastases was shorter for patients with synchronous systemic and CNS relapse vs. patients with isolated CNS relapse (4.3 months [95% CI: 0.4, 11.6] vs. 15.6 months [95% CI: 7.3, 31.8], *P* = .003, Figure 2D). Details of subsequent therapies received in patients with CNS metastases are shown in supplemental Table 2.

## Discussion

In this retrospective multi-center study, 16.6% of patients with unresectable LA-NSCLC treated with chemoRT followed by durvalumab developed CNS metastases, higher than in published literature.^7^ Most CNS metastases developed within a year of starting durvalumab. Increasing stage among patients with *de novo* LA-NSCLC was associated with development of CNS metastases. Patients with CNS metastases had shorter survival.

Improved understanding of drivers of CNS metastases is critical. Prior studies examining recurrence patterns in this patient population have been limited by nonuniform receipt of durvalumab. Nonetheless, low PD-L1 tumor proportion score (TPS) and higher nodal stage are associated with risk of any systemic recurrence.^14^ Another study showed low PD-L1 TPS and squamous histology increased risk of locoregional recurrence while female sex and *EGFR* mutations increased risk of distant recurrence.^7^ These risk factors did not associate with risk of CNS metastases, specifically, in our cohort that universally received durvalumab, consistent with current US standard of care. Whether previously identified risk factors are not relevant to CNS metastases or whether durvalumab may abrogate the risk is unclear.

Durvalumab, like other PD(L)1 antibodies,^15^ may be active against micrometastatic CNS disease as suggested by the placebo group in PACIFIC experiencing nearly twice the CNS metastases as the durvalumab group. We found that MRI to IO interval >3 months was associated with risk of CNS metastases, suggesting delayed immunotherapy may be 1 driver of CNS metastases. Similarly, fewer durvalumab doses was associated with CNS metastases, though interpretation of this finding is challenging since earlier progression could lead to earlier discontinuation. Indeed, as no repeat brain MRI was required between chemoRT and durvalumab, it is possible early CNS metastases which prompted durvalumab discontinuation actually developed prior to durvalumab exposure, and points to the potential role for repeat brain imaging after completion of chemoRT to guide treatment.

Our study adds to the literature using updated staging from version 8 of the *Staging Manual in Thoracic Oncology* which reflects current practice in the US. The PACIFIC trial utilized version 7. While T and N stages were not associated with CNS metastases in our dataset, overall stage (IIIB/C vs. IIIA) among patients with *de novo* disease was. Other strengths of our study include examination of risk factors for CNS metastases, specifically, as opposed to any recurrence, and the multi-center nature with a larger patient cohort than previous studies.^6,10^

Limitations include inconsistent genomic profiling, which may miss genomic drivers of CNS metastases, and retrospective nature, meaning cases of CNS metastases were possibly missed due to incomplete follow-up data. Additionally, selection bias may exist because these patients were managed only at academic centers. Furthermore, cases of occult CNS metastases may have been missed due to current real-world CNS imaging practices. Also, no survival analyses based on CNS metastases management strategy were performed due to small sample size, though this would inform interpretation of OS data.

CNS metastases remain a significant challenge and strategies to address them are needed. Screening for occult CNS disease after initial staging is not currently recommended in NCCN guidelines. However, our study demonstrates that frequency of CNS metastases is higher than previously estimated, they tend to occur early, and survival is superior in isolated cases. This combination of factors suggests a possible opportunity for early detection and intervention, especially among the highest risk population (i.e. *de novo* stage IIIB and IIIC), which may lead to improved clinical outcomes. We believe the time is right for well-designed clinical trials testing such a strategy.

## Supplementary Material

1

## Figures and Tables

**Table 1:** Multivariable analysis for risk of development of central nervous system metastases among patients with locally advanced nonsmall cell lung cancer managed with concurrent chemoradiation followed by durvalumab in overall cohort (*n* = 193) and those presenting with *de novo*, nonlocally recurrent disease only (*n* = 155)

Parameter		Hazard Ratio	95% Hazard Ratio Confidence Limits		*P*-value
Overall cohort (*n* = 193)					
Time between baseline brain MRI and start of durvalumab	>3 months vs. ≤3 months	2.66	1.01	7.02	.0475
Durvalumab doses		0.93	0.89	0.97	.0026
Age		0.99	0.95	1.02	.4860
Cohort with *de novo* locally advanced disease (*n* = 155)					
Time between baseline brain MRI and start of durvalumab	>3 months vs. ≤3 months	4.46	1.33	15.00	.016
Durvalumab doses		0.93	0.88	0.978	.005
Stage	IIIB+IIIC vs. IIIA	3.85	1.44	10.35	.007

Abbreviations: MRI = magnetic resonance imaging.

## Clinical Practice Points

- In this multi-center study of patients with unresectable locally advanced non-small cell lung cancer undergoing concurrent chemoradiation followed by durvalumab, central nervous system (CNS) metastases developed in 16.6% of patients, higher than previously reported in the literature.
- Higher baseline stage (IIIB/C vs. IIIA) and an interval of >3 months from staging brain MRI to initiation of durvalumab were associated with an increased risk of developing CNS metastases.
- Overall survival was shorter for patients who developed CNS metastases. Patients with synchronous systemic and CNS metastases had a particularly short overall survival.

## References

[1] Wong MCS, Lao XQ, Ho K-F, Incidence and mortality of lung cancer: global trends and association with socioeconomic status. Sci Rep. 2017;7:14300. 29085026 10.1038/s41598-017-14513-7 PMC5662733

[2] Casal-Mouriño A, Ruano-Ravina A, Lorenzo-González M, Epidemiology of stage III lung cancer: frequency, diagnostic characteristics, and survival. Transl Lung Cancer Res. 2020;10:506–518. 10.21037/tlcr.2020.03.40 PMC7867742 33569332

[3] Antonia SJ, Villegas A, Daniel D, Durvalumab after chemoradiotherapy in stage III non–Small-cell lung cancer. N Engl J Med. 2017;377:1919–1929. 28885881 10.1056/NEJMoa1709937

[4] Antonia SJ, Villegas A, Daniel D, Overall survival with Durvalumab after chemoradiotherapy in stage III NSCLC. N Engl J Med. 2018;379:2342–2350. 30280658 10.1056/NEJMoa1809697

[5] Ali A, Goffin JR, Arnold A, Survival of patients with non-small-cell lung cancer after a diagnosis of brain metastases. Curr Oncol. 2013;20:e300–e306. 23904768 10.3747/co.20.1481 PMC3728058

[6] Cagney DN, Martin AM, Catalano PJ, Incidence and prognosis of patients with brain metastases at diagnosis of systemic malignancy: a population-based study. Neuro Oncol. 2017;19:1511–1521. 28444227 10.1093/neuonc/nox077 PMC5737512

[7] Kishi N, Matsuo Y, Shintani T, Recurrence patterns and progression-free survival after chemoradiotherapy with or without consolidation durvalumab for stage III non-small cell lung cancer. J Radiat Res. 2023;64:142–153. 36149029 10.1093/jrr/rrac057 PMC9855316

[8] Ernani V, Stinchcombe TE. Management of brain metastases in non–Small-cell lung cancer. J Oncol Pract. 2019;15:563–570. 31715122 10.1200/JOP.19.00357 PMC7098835

[9] Patchell RA, Tibbs PA, Walsh JW, A randomized trial of surgery in the treatment of single metastases to the brain. N Engl J Med. 1990;322:494–500. 2405271 10.1056/NEJM199002223220802

[10] Brown PD, Ballman KV, Cerhan JH, Postoperative stereotactic radiosurgery compared with whole brain radiotherapy for resected metastatic brain disease (NCCTG N107C/CEC·3): a multicentre, randomised, controlled, phase 3 trial. Lancet Oncol. 2017;18:1049–1060. 28687377 10.1016/S1470-2045(17)30441-2 PMC5568757

[11] Mahajan A, Ahmed S, McAleer MF, Post-operative stereotactic radiosurgery versus observation for completely resected brain metastases: a single-centre, randomised, controlled, phase 3 trial. Lancet Oncol. 2017;18:1040–1048. 28687375 10.1016/S1470-2045(17)30414-X PMC5560102

[12] Preti BTB, Sanatani MS, Breadner D, Real-world analysis of Durvalumab after chemoradiation in stage III non-small-cell lung cancer. Curr Oncol. 2023;30:7713–7721. 37623040 10.3390/curroncol30080559 PMC10453685

[13] Girard N, Bar J, Garrido P, Treatment characteristics and real-world progression-free survival in patients with unresectable stage III NSCLC who received Durvalumab after chemoradiotherapy: findings from the PACIFIC-R study. J Thorac Oncol. 2023;18:181–193. 36307040 10.1016/j.jtho.2022.10.003

[14] Takahara Y, Tanaka T, Ishige Y, Early recurrence factors in patients with stage III non-small cell lung cancer treated with concurrent chemoradiotherapy. Thorac Cancer. 2022;13:3451–3458. 36281714 10.1111/1759-7714.14704 PMC9750816

[15] Goldberg SB, Gettinger SN, Mahajan A, Pembrolizumab for patients with melanoma or non-small-cell lung cancer and untreated brain metastases: early analysis of a non-randomised, open-label, phase 2 trial. Lancet Oncol. 2016;17:976–983. 27267608 10.1016/S1470-2045(16)30053-5 PMC5526047

