# Supplementary material for: Brief Report: Central Nervous System Metastases After Chemoradiation Followed by Durvalumab for Unresectable Locally Advanced Nonsmall Cell Lung Cancer
Source: Clin Lung Cancer. Author manuscript; Available in PMC 2026 May 7. (PMC13150787; doi:10.1016/j.cllc.2025.07.002)
Supplement: 1 [file NIHMS2169362-supplement-1.pdf]

Supplementary material

Table S1 and Table S2

Table S1 Clinical and disease-related characteristics of patients.

Characteristic	Total Cohort (n=193)
Age, median (range)	64 (37 - 86)
Female sex, n (%)	85 (44.0%)
Race, n (%)	
White	159 (82.4%)
Black/African American	31 (16.1%)
Other/Not Reported	3 (1.6%)
Smoking, n (%)	
Current	60 (31.1%)
Former	121 (62.7%)
Unknown	12 (6.2%)
ECOG PS at chemoRT, n (%)	
0	29 (15.0%)
1	121 (62.7%)
2	30 (15.5%)
3+	3 (1.6%)
Not Reported	10 (5.2%)
Histology, n (%)	
Nonsquamous	96 (49.7%)
Squamous	96 (49.7%)
Not Reported	1 (0.5%)
T stage, n (%)	
T1	44 (22.8%)
T2	42 (21.8%)
T3	39 (20.2%)
T4	55 (28.5%)
Tx/Not Reported	13 (6.7%)
N stage, (%)	
N0	36 (18.7%)
N1	22 (11.4%)
N2	91 (47.1%)
N3	38 (19.7%)
Not Reported	6 (3.1%)
Locally recurrent, n (%)	38 (19.7%)
De novo disease, n (%)	155 (80.3%)
Stage IIIA	73 (47.1%)
Stage IIIB	57 (36.8%)
Stage IIIC	16 (10.3%)
Not reported	9 (5.8%)
PD-L1 by TPS, n (%)	
<1%	58 (30.1%)
1-49%	62 (32.1%)
50+%	56 (29.0%)
Not Reported	17 (8.8%)
Mutation status, n (%)*	
TP53	57 (29.5%)

(*continued on next page*)

Table S1 (*continued*)

KRAS	29 (15.0%)
STK11	8 (4.1%)
EGFR	1 (0.5%)
Genomics not performed/reported	46 (23.8%)
Locally recurrent at chemoRT, n (%)	38 (19.7%)
ChemoRT regimen, n (%)	171 (88.6%)
Carboplatin-based	
Cisplatin-based	22 (11.4%)
Median duration from completion chemoRT to first dose of durvalumab, months (IQR)	1.12 (0.72 – 1.41)
Median time from baseline brain MRI to start of durvalumab, months (IQR)	3.35 (2.83– 4.11)
Median doses of durvalumab, n (range)	11 (1 – 27)

*Mutations non-mutually exclusive.
Abbreviations: ChemoRT=concurrent chemoradiation, CNS=central nervous system, ECOG PS=Eastern Cooperative Oncology Group performance status, IQR=Interquartile range, MRI=magnetic resonance imaging, PD-L1=programmed cell death ligand 1, TPS=tumor proportion score.

Table S2 **Subsequent therapies in patients with CNS metastases.**

Patients with CNS metastases	32 (100.0%)
Received subsequent anticancer treatment	27 (84.4%)
Patients with isolated CNS metastases	**20 (62.5%)**
Received subsequent anticancer therapy	18 (95.0%
CNS-directed therapy	
SRS alone	8 (40.0%)
Pre-operative SRS + surgical resection	8 (40.0%)
Surgical resection alone	2 (10.0%)
Patients with synchronous CNS and systemic metastases	**12 (37.5%)**
Received subsequent anticancer therapy	9 (75.0%)
Systemic therapy	
Chemoimmunotherapy	4 (33.3%)
Chemotherapy alone	3 (25.0%)
Immunotherapy alone	1 (8.3%)
Targeted therapy (selpercatinib)	1 (8.3%)
CNS-directed therapy	
SRS alone	3 (25.0%)
Pre-operative SRS + surgical resection	1 (8.3%)

Abbreviations: CNS=central nervous system, SRS=stereotactic radiosurgery.
